# Multi-Timescale Drowsiness Characterization Based on a Video of a Driver’s Face

**DOI:** 10.3390/s18092801

**Published:** 2018-08-25

**Authors:** Quentin Massoz, Jacques G. Verly, Marc Van Droogenbroeck

**Affiliations:** Department of Electrical Engineering and Computer Science, Faculty of Applied Science, University of Liège, B-4000 Liège, Belgium; jacques.verly@uliege.be (J.G.V.); m.vandroogenbroeck@uliege.be (M.V.D.)

**Keywords:** drowsiness, driver monitoring, multi-timescale, eye closure dynamics, psychomotor vigilance task, reaction time, convolutional neural network

## Abstract

Drowsiness is a major cause of fatal accidents, in particular in transportation. It is therefore crucial to develop automatic, real-time drowsiness characterization systems designed to issue accurate and timely warnings of drowsiness to the driver. In practice, the least intrusive, physiology-based approach is to remotely monitor, via cameras, facial expressions indicative of drowsiness such as slow and long eye closures. Since the system’s decisions are based upon facial expressions in a given time window, there exists a trade-off between accuracy (best achieved with long windows, i.e., at long timescales) and responsiveness (best achieved with short windows, i.e., at short timescales). To deal with this trade-off, we develop a multi-timescale drowsiness characterization system composed of four binary drowsiness classifiers operating at four distinct timescales (5 s, 15 s, 30 s, and 60 s) and trained jointly. We introduce a multi-timescale ground truth of drowsiness, based on the reaction times (RTs) performed during standard Psychomotor Vigilance Tasks (PVTs), that strategically enables our system to characterize drowsiness with diverse trade-offs between accuracy and responsiveness. We evaluated our system on 29 subjects via leave-one-subject-out cross-validation and obtained strong results, i.e., global accuracies of 70%, 85%, 89%, and 94% for the four classifiers operating at increasing timescales, respectively.

## 1. Introduction

Drowsiness is defined as the intermediate, physiological state between wakefulness and sleep. It is associated with a difficulty to stay awake, a strong desire to fall asleep, and is characterized by impairments of performance, both cognitive [1,2] and motor [3,4]. While driving, drowsiness becomes a serious traffic safety hazard that leads to the death of thousands of drivers each year [5]. A solution is to develop automatic, real-time drowsiness characterization systems that aim at preventing these accidents by issuing accurate and timely (i.e., responsive) drowsiness warnings to the driver, or to a semi-autonomous driving system. In operational settings, these drowsiness characterization systems are generally based on driving performance (e.g., wheel steering, braking, and line crossing) and/or operator physiology (e.g., brain signals, heart rate, and facial expressions). Overall, the systems based on facial expressions have the significant advantages of being mostly independent of applications and vehicle types, less sensitive to external conditions (e.g., weather, and traffic), and non-intrusively implementable with remote sensors such as cameras. Among the facial expressions, the dynamics of eye closure is recognized as a strong and reliable physiological indicator of drowsiness [6,7]. For example, blinks become unconsciously slower and longer when the level of drowsiness increases.

In the scientific literature, systems typically make use of eye closure dynamics by averaging blink-related features (e.g., blink duration) over a time window of fixed length (e.g., one minute). However, systems using this strategy suffer from a trade-off between accuracy and responsiveness. Indeed, a system based on a short time window (of eye closure dynamics) will be very responsive to brief episodes of drowsiness such as lapses and microsleeps, but it will not estimate drowsiness with high accuracy, whereas a system based on a long time window will be more accurate, but less responsive. Ideally, drowsiness characterization systems should be both accurate and responsive.

**Figure 1:**
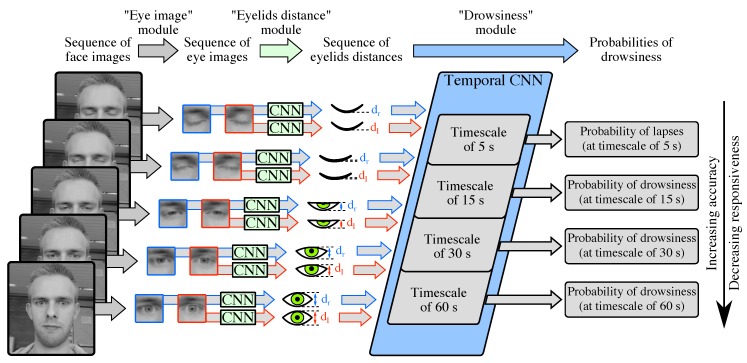
Overview of our multi-timescale drowsiness characterization system operating on any given 1-min sequence of face images. First, from each face image, the “eye image” module produces two eye images (left and right) via off-the-shelf algorithms. Second, from each eye image, the “eyelids distance” module produces the eyelids distance via a convolution neural network (CNN). Third, from the 1-min sequence of eyelids distances and via a temporal CNN, the “drowsiness” module (1) extracts features related to the eye closure dynamics at four timescales, i.e., the four most-recent time windows of increasing lengths (5 s, 15 s, 30 s, and 60 s), and (2) produces four probabilities of drowsiness of increasing accuracy, but decreasing responsiveness.

With the goal of satisfying both accuracy and responsiveness, we present a novel multi-timescale drowsiness characterization system that is data-driven, automatic, real-time, and generic. Our system extracts, via convolutional neural networks (CNNs), data-driven features related to eye closure dynamics at four timescales, i.e., four time windows of increasing lengths (5 s, 15 s, 30 s, and 60 s) and all extending up to the present, so as to infer four binary Levels of Drowsiness (LoDs). We design a novel multi-timescale ground truth of drowsiness in such a manner that (1) an LoD inferred at a low timescale is an early and responsive, but noisy estimate of drowsiness, and (2) an LoD inferred at a high timescale is an accurate, but less responsive estimate of drowsiness. More specifically, to obtain such multi-timescale ground truth, we produce four binary ground-truth LoDs (one per inferred LoD) based on the median values, computed over time windows of increasing lengths, of the reaction times (RTs) performed during standard Psychomotor Vigilance Tasks (PVTs). In such a manner, our system produces, from any 1-min sequence of face images, four LoDs with diverse trade-offs between accuracy and responsiveness. Our system consists of a succession of three processing modules. Figure 1 depicts the architecture of our system and its three modules.

The remainder of this article is organized as follows. Section 2 presents a background concerning the field of automatic, real-time characterization of drowsiness, including the related systems of other studies, and lists our main contributions to this field. Section 3 details the data we collected, and the multi-timescale ground truth of drowsiness we produced to develop our multi-timescale drowsiness characterization system. Section 4 details the architecture of our system. Section 5 details the training of our system. Section 6 evaluates the performance of our system. Section 7 compares by proxy the performance of our system with those of systems of other studies. Section 8 investigates the combination of the four binary LoDs into a single LoD, which is more convenient for operational uses. Section 9 concludes this article.

## 2. Background on Automatic, Real-Time Characterization of Drowsiness

Drowsiness is a complex physiological state, the continuous level of which is not a precisely and numerically defined quantity that can be directly measured. Therefore, the practical way to quantify drowsiness is by characterizing it based on measurable indicators of drowsiness. We distinguish two types of indicators: the subjective ones (i.e., subjective questionnaires), and the objective ones (i.e., changes in physiology, and impairments of performance). The choice of which indicators to use depends on whether they will be used (1) as inputs to the characterization system, or (2) to produce a ground truth used to train the system and/or evaluate its performance.

As inputs, the only adequate indicators are the objective ones. Indeed, subjective questionnaires (e.g., the Karolinska Sleepiness Scale [8]) require the driver to briefly self-assess his/her drowsiness and to report it manually, which would defeat the purpose of an automatic system. Performance-based indicators, such as responsiveness performance [9] and driving performance [10], are not ideal as the former requires a secondary task to be performed (which would hinder the main task of driving), and the latter is sensitive to many variables different from drowsiness (e.g., vehicle type, traffic, weather, road condition, and road type). On the contrary, physiology-based indicators are mostly independent of application and vehicle type, and less sensitive to external conditions. Physiology-based indicators such as changes in polysomnography signals [8], heart rate [11], and skin conductance [12] require, to be measured, electrodes in contact with the skin, which is invasive and thus non-practical. However, physiology-based indicators such as changes in facial expressions can be measured non-intrusively with remote sensors such as cameras. Among the facial expressions, the eye closure dynamics is recognized as a strong and reliable indicator of drowsiness [6,7]. Furthermore, considering that blinks naturally occur once every few seconds, eye closure dynamics constitutes a regular stream of insights about the physiological impacts of drowsiness. This inherent attribute makes the eye closure dynamics an indicator of choice to base automatic, real-time drowsiness characterization systems upon.

To produce a ground truth, the scientific community has yet to reach a clear consensus on which indicator is best to use [13]. Indeed, understanding drowsiness, i.e., its causes, dynamics, and effects, is still an active and challenging field of research. The scientific literature generally quantizes the ground truth of drowsiness as a discrete LoD taking *N* distinct integer values (with N≥2) and annotated based on various indicators of drowsiness. The ground-truth LoD can be self-annotated by subjects in terms of a subjective questionnaire [14,15], marked positive when line crossings occur in a driving simulator [16] or on real roads [17], annotated by trained experts by visually looking for physiological indicators of drowsiness in the brain signals [17,18] or in the face video [19,20], or non-spontaneously acted out by subjects according to a pre-defined, given script [21,22,23].

In the context of developing automatic, real-time drowsiness characterization systems, defining and obtaining such ground-truth LoD are both essential steps. Indeed, the task of interpreting facial expressions over time so as to characterize a physiological state such as drowsiness is a complex and challenging one. In particular, the procedure that a human would carry out in order to perform such interpretation may not be easily implementable as an automatic algorithm. Therefore, such systems generally use machine learning models trained in a supervised manner, which requires a ground truth to be available. In practice, these learned systems typically adopt the cascade structure that consists in first (1) extracting an intermediate representation, e.g., a vector of features [14,15,16,17,18,19] or a sequence of features [20,21,22], and then (2) characterizing drowsiness, as defined by the selected type of ground truth. Note that these features generally consist of standard measures of objective indicators, such as the percentage of eye closure (PERCLOS) and the standard deviation of lateral position (SDLP). Compared to “black box”, end-to-end systems [23], systems with a cascade structure have the key properties of having greater interpretability, modularity, and data efficiency. Interpretability facilitates the explanation of the system’s decisions, which is of great importance since wrong decisions—although intrinsically unavoidable—should be explainable to humans for (1) the legal and public acceptance of the technology, and for (2) its future improvements, in particular for safety-related applications where human lives are at stake. Modularity enables (online and offline) adaptations to how the intermediate representation is extracted so as to perform better in real-life, operational settings, while being able to keep the characterization of drowsiness as is, i.e., as developed in laboratory settings. Data efficiency enables the system to obtain better performance with an equivalent, limited amount of data.

The scientific literature provides several possible algorithms to extract the intermediate representation, and models to characterize drowsiness. For extracting the intermediate representation, algorithms consist of proprietary softwares [14,17,18], face landmarks alignment [16,20,21], thresholds on the first derivative of the electro-oculogram (EOG) signal [15], adaptative image filters and statistical fitting [19], or a pre-trained CNN [22] such as the VGG-16 one [24]. For characterizing drowsiness, models consist of logistic regression [16,17], support vector machine (SVM) [15], artificial neural network (ANN) [14,15,19], hidden Markov model (HMM) [20,21], long-short term memory (LSTM) network smoothed by a temporal CNN [22], or end-to-end 3D-CNN [23]. Table 1 lists the design choices made by others in the field of automatic, real-time drowsiness characterization systems based on faces images, and compares them to the ones of our system (in bold).

Existing systems are limited by the inherent trade-off between accuracy and responsiveness, which stems from the use of a single time window of a fixed length. In this article, we propose a multi-timescale system, using multiple time windows of different lengths, so as to infer four binary LoDs with diverse trade-offs between accuracy and responsiveness. Our main contributions to the field of automatic, real-time drowsiness characterization are as follows:

- we present a multi-timescale system to deal with the trade-off between accuracy and responsiveness;
- we introduce an appropriate multi-timescale ground truth to train such a multi-timescale system, which is based on objective, performance-based indicators, i.e., the RTs performed during PVTs;
- we use the sequence of raw eyelids distances (produced by a CNN, trained from scratch) as the intermediate representation, which we show to lead to strong results when processed by a multi-timescale temporal CNN;
- we adopt a strict, rigorous evaluation scheme (i.e., leave-one-subject-out cross-validation), and compare, by proxy, the performance of our system with the performances of systems of other studies;
- we make our drowsiness dataset, code, and trained models available (see details in Appendix).

## 3. Our Drowsiness Dataset

We collected data from 35 young, healthy subjects (21 females and 14 males) with ages of 23.3 ± 3.6 years (mean ± standard deviation), and free of drug, alcohol, and sleep disorders. The subjects were acutely deprived of sleep for up to 30 h over two consecutive days, and were forbidden to consume any stimulants. During this period, the subjects performed three 10-min PVTs: PVT1 took place at 10–11 a.m. (day 1), PVT2 at 3:30–4 a.m. (day 2), and PVT3 at 12–12:30 p.m. (day 2). The PVTs were performed in a quiet, isolated laboratory environment without any temporal cues (e.g., watch or smartphone). The room lights were turned off for PVT2 and PVT3. At the end of the study, we strongly advised the subjects not to drive home by themselves, and we offered them alternative transportation solutions for free when necessary. The study protocol was approved by the Ethics Committee of the University of Liège.

We adopted the PVT implementation proposed by Basner and Dinges [9], where the subject is instructed to react as fast as possible (via a response button) to visual stimuli occurring on a computer screen at random intervals (ranging from 2 to 10 s). In order to obtain more variability in head pose and gaze direction, we made the visual stimuli occur randomly among five positions on the screen, i.e., at its center and at its four corners. During each 10-min PVT, we recorded the RTs (in milliseconds), as well as the near-infrared face images (at 30 frames per second) via the Microsoft Kinect v2 sensor (Microsoft Corporation, Redmond, WA, USA).

Due to some technical issues, only 88 PVTs (from 32 subjects, 20 females and 12 males) turned out to be usable. Out of them, we only included 82 PVTs (from 29 subjects, 18 females and 11 males) in the present study, because the PVT1 data (which are necessary for normalizing RTs in Section 3.1) were missing for 3 subjects. We make the full dataset (of 88 PVTs) available alongside the present article (see details in Appendix). However, for reasons of privacy, we only provide, in the dataset, (1) the RTs and (2) the intermediate representations of our system (i.e., the sequences of eyelids distances), and not the near-infrared face images.

### 3.1. Inter-Subject Normalization of the Reaction Times (RTs)

The reaction time achieved by a subject depends on various factors including drowsiness, time-on-task (i.e., fatigue), and individual skills. Drowsiness is the state that we wish to characterize, time-on-task is considered of minor impact as it remains below 10 minutes, and individual skills can be mitigated by inter-subject normalization. Considering that the reciprocal of the RT (i.e., the reaction speed) of an individual follows relatively well a normal distribution [25], we normalize each RT from each subject according to

(1) $$x^{\prime }={\left(\frac{1}{x}-\mu_{k}+\frac{1}{29}\sum_{i=1}^{29}\mu_{i}\right)}^{-1},$$

where *k* is the subject index, *x* is a recorded RT from subject *k*, $x^{\prime }$ is the corresponding normalized RT for subject *k*, and $\mu_{k}$ is the mean of the reciprocal of all RTs recorded during PVT1 of subject *k*. This normalization shifts the RTs distribution of a subject in an alert state (i.e., in the first morning, during PVT1) to the population average (estimated from the 29 subjects).

### 3.2. Generation of the Multi-Timescale Ground Truth of Drowsiness

In this article, we want to develop a drowsiness characterization system operating both at long timescales (leading to accurate estimation of drowsiness) and at short timescales (leading to responsive estimation of drowsiness). Therefore, we need to produce the appropriate ground-truth LoDs of increasing accuracy and of decreasing responsiveness. Given that drowsiness is characterized by impairments of performance, i.e., overall longer RTs while performing a PVT, a ground-truth LoD could be generated by thresholding either (1) a single RT, which is perfectly time-localized (resulting in a responsive, but noisy estimate of drowsiness) or (2) a metric computed from a set of RTs within a time window (resulting in a more accurate, but less responsive estimate of drowsiness).

Accordingly, we define four metrics of performance, which we call “median RTs”, denoted by $m_{i}$ with i∈{1,2,3,4}. The first median RT, $m_{1}$, corresponds to a single RT that either (1) occurs within the [−1s,+1s] time window or (2) is a linear interpolation between the previous RT and the next RT. The other median RTs, $m_{2}$, $m_{3}$, and $m_{4}$, are computed as the harmonic means (equivalent to the medians of the reciprocal normal distributions) of the RTs that occur within the [−15s,+5s], [−30s,+5s], and [−60s,+5s] time windows, respectively. Each median RT can be considered as being a continuous signal that varies in time at a specific timescale, induced by its corresponding sliding time window. These time windows are allowed to be non-causal since they are used for producing the ground-truth LoDs, and thus not for operational use.

By thresholding these four median RTs, we obtain four binary ground-truth LoDs, each varying at a distinct timescale, and each associated with a ground-truth likelihood score of drowsiness (loosely referred to as a probability of drowsiness from here on), denoted by $p_{i}$ and defined as

(2) $$p_{i}=\left\{\begin{array}{cc} \begin{array}{c} 0\\ 0.5\\ 1 \end{array} & \begin{array}{c} \mathrm{if}m_{i}\leq 400\mathrm{ms}\\ \mathrm{if}m_{i}\in ]400,500[\mathrm{ms}\\ \mathrm{if}m_{i}\geq 500\mathrm{ms} \end{array} \end{array}\right.,\;\mathrm{for}\;\mathrm{each}\;i\in \left\{1,2,3,4\right\}.$$

The above thresholds of 400 ms and 500 ms were chosen empirically, yet pertinently. Indeed, the threshold of 400 ms corresponds to about the 98–99^th^ percentile of the distribution of $m_{4}$ during PVT1 (i.e., in non-sleep deprived conditions), whereas the threshold of 500 ms corresponds to the value above which a RT (such as $m_{1}$) is conventionally interpreted as a lapse [3,9]. From here on, each ground-truth LoD is referenced either by its index (1 to 4), or by the timescale at which the classifier estimating it operates (5 s, 15 s, 30 s, and 60 s, respectively).

## 4. Architecture of Our Multi-Timescale Drowsiness Characterization System

Our drowsiness characterization system is composed of three modules operating in cascade: the “eye image” module, the “eyelids distance” module, and the “drowsiness” module.

### 4.1. “Eye Image” Module

**Figure 2:**
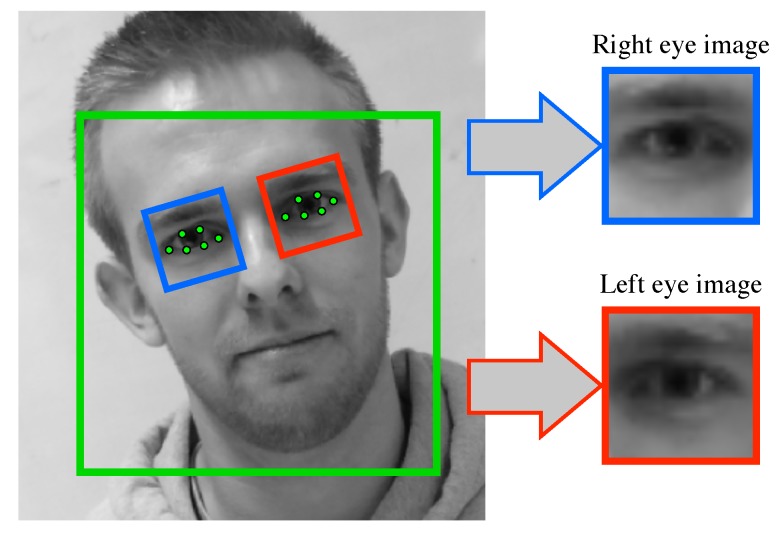
Illustration of the steps of the “eye image” module. In succession, we detect the face (green square), we align the eyelids landmarks (green dots), and we geometrically extract the right eye image (blue square) and the left eye image (red square) with a common size of 24×24 pixels.

This module is composed of off-the-shelf algorithms and extracts, for each frame and for each eye, an eye image of size 24×24 pixels, this in four successive steps. First, we detect the face region using the OpenCV [26] implementation of the Viola and Jones algorithm [27]. Second, within the detected face region, we localize 68 face landmarks using the dlib [28] implementation of the Kazemi and Sullivan algorithm [29]. Third, from the 12 eyelids landmarks, we compute the eye center positions of the right and left eye, $c_{r}$ and $c_{l}$, respectively, and the rotation angle needed to align them horizontally, α. Fourth (and last), we extract the right and left eye images using affine warping so as to obtain a right (respectively left) eye image centered on $c_{r}$ (respectively $c_{l}$), rotated by an angle of α around $c_{r}$ (respectively $c_{l}$), scaled at 24% of the face region width (from the first step), and with size of 24×24 pixels. Figure 2 depicts the extraction of both eye images.

### 4.2. “Eyelids Distance” Module

This module is a spatial CNN taking, as input, a grayscale eye image, and producing, as output, an estimate of the eyelids distance (i.e., a real number) in pixels (referenced in the eye image, not in the original frame). The architecture of the module is very similar to the VGGNet architecture [24].

**Figure 3:**
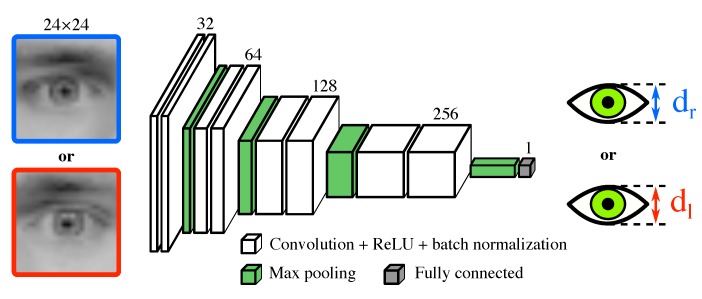
Architecture of the “eyelids distance” module. The CNN produces an estimate of the right (or left, respectively) eyelids distance (i.e., a real number) from the right (or left, respectively) eye image of size 24×24 pixels. Note that one can process both eye images simultaneously in a batch of size 2.

The eye image is sequentially processed by (1) eight 3×3 convolutional layers (stride of 1, padding of 2, depths of 32, 32, 64, 64, 128, 128, 256, and 256, respectively, followed by ReLU then batch normalization [30]) interspersed with three 2×2 max pooling layers (padding of 2) positioned after every two convolutional layers, (2) a global max pooling, and (3) a fully connected layer (depth of 1) so as to output the eyelids distance, i.e., a real number. Figure 3 depicts the architecture of this module.

### 4.3. “Drowsiness” Module

**Figure 4:**
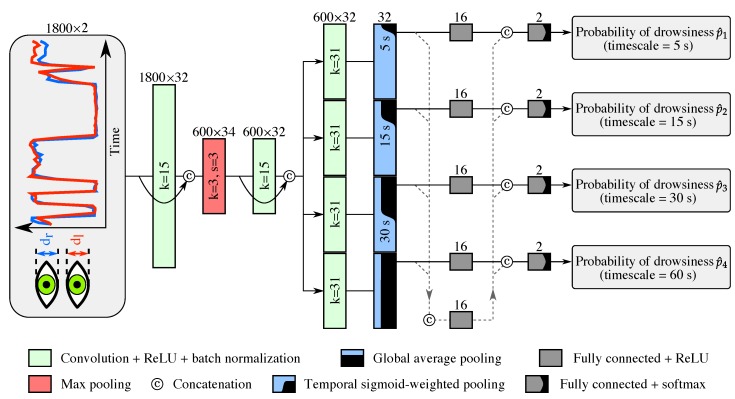
Architecture of the “drowsiness” module. The temporal CNN processes a 1-min sequence of eyelids distances using multiple time windows extending up to the present (via global pooling) to characterize drowsiness at multiple timescales.

This module is a temporal CNN taking, as input, a 1-min sequence of eyelids distances related to both eyes (1800×2 values, at a framerate of 30 frames per second), and producing, as output, an estimate of the four probabilities of drowsiness, denoted by ${\hat{p}}_{i}$, varying each at a different timescale (as defined in Section 3.2). The processing is depicted in Figure 4, and is as follows.

First, the module processes the input sequence with two temporal convolutional layers (depth of 32, receptive field of 15, stride of 1, padding of 7, followed by ReLU then batch normalization) separated by a max pooling layer (receptive field *k* of 3, and stride *s* of 3). These two convolutional layers are densely connected [31], meaning that their outputs are concatenated with their inputs via a skip connection, leading to output sequences with dimensions of 34 and 66, respectively.

Second, the module forwards the resulting sequence (with depth of 66) to four branches, each tasked to produce one of the four estimated probabilities of drowsiness ${\hat{p}}_{i}$. Each branch consists of (1) a temporal convolutional layer (depth of 32, receptive field *k* of 31, stride *s* of 1, padding of 15, followed by ReLU then batch normalization, and without skip connection), (2) a global pooling layer (different for each branch, see below), (3) a first fully connected layer (depth of 16, and followed by ReLU), and (4) a last fully connected layer (depth of 2) followed by the softmax function.

Because the ground-truth LoD signal varies rapidly at a low timescale, the estimation of drowsiness should be mostly based on a short time window so as to be responsive to sudden changes in the eye closure dynamics. Therefore, the global pooling of the first three branches focus their attention over the recent past of varying length $n_{0}$ (of 5 s, 15 s, and 30 s for the timescales of 5 s, 15 s, and 30 s, respectively) via a “temporal sigmoid-weighted pooling” layer, represented in Figure 4, and defined as

(3) $$a\left(n_{0}\right)=\sum_{n=1}^{600}\frac{\sigma \left(\frac{3}{2}\left(n-600+10n_{0}\right)\right)}{\sum_{k=1}^{600}\sigma \left(\frac{3}{2}\left(k-600+10n_{0}\right)\right)}v_{n},$$

where a is the output feature vector, $v_{n}$ is the feature vector at the *n*th position in the input sequence, σ(x) is the sigmoid function expressed as ${\left(1+e^{-x}\right)}^{-1}$, and $n_{0}$ is the cut-off time (expressed in seconds) of the attention weights. We chose the sigmoid function to have the temporal weigths decrease sharply, yet smoothly, at $n_{0}.$ The global pooling of the fourth branch (timescale of 60 s) corresponds to a global average pooling.

Furthermore, we add what we call “multi-timescale context” to each branch: the outputs of the global pooling layer of each branch are concatenated together, processed by a fully connected layer (depth of 16, and followed by ReLU), and then concatenated back into each branch with the output of their respective first fully connected layer. This is equivalent to adding dependencies between the branches, which we will show to be crucial to obtain strong performance for estimating drowsiness at low timescales.

## 5. Training of Our System

We trained the “eyelids distance” module and the “drowsiness” module sequentially. Implementation details and links for code are provided in Appendix.

### 5.1. “Eyelids Distance” Module

We built a dataset specifically for training and evaluating the performance of this module. This dataset consists of the Multi-PIE (MPIE) face dataset [32] augmented with a small subset of near-infrared face images (834) from our drowsiness dataset (denoted DD). We chose the MPIE dataset because of its variety in subjects, illumination conditions, head poses (from frontal to near-profile head poses), and types of eyeglasses (when present).

For each face image (of both sub-datasets), we extracted two eye images, i.e., one for each eye, by making use of the 68 manually-annotated face landmarks. For each eye image, we computed the ground-truth eyelids distance (i.e., the target) as the average of the two inter-eyelid Euclidean distances (referenced in the eye image) between the two face landmarks positioned on the upper eyelid, and the two on the lower eyelid.

We split this dataset into a training set, a validation set, and a test set intended for training the model parameters, validating its hyper-parameters (via random search), and evaluating its performance, respectively. Table 2 contains the number of subjects and samples in these three sets, and from each of the two sub-datasets (MPIE or DD).

We trained the “eyelids distance” module with the Mean Squared Error (MSE) loss function using the RMSProp [33] optimization routine with a smoothing constant α of 0.9886, a batch size of 32, and a learning rate of 0.001428. We normalized the eye images by subtracting the average pixel value computed from the training set. We doubled the number of samples of the training, validation, and test sets by horizontally flipping every eye images. We performed no other data augmentation.

### 5.2. “Drowsiness” Module

We trained this module with the average of four binary relative entropies, each associated with one of the four probabilities of drowsiness. The loss function is given for one sample by

(4) $$L\left(\hat{p},p\right)=\frac{-1}{4}\sum_{i=1}^{4}\left[p_{i}\ln\left(\frac{{\hat{p}}_{i}}{p_{i}}\right)+\left(1-p_{i}\right)\ln\left(\frac{1-{\hat{p}}_{i}}{1-p_{i}}\right)\right],$$

where ${\hat{p}}_{i}$ is the *i*th estimated probability of drowsiness produced by our model, and $p_{i}$ is the *i*th ground-truth probability of drowsiness defined in Equation (2).

Considering the limited number of subjects (29), we trained 29 models following a leave-one- subject-out cross-validation strategy of 29 folds. Each fold consists of a training set of 23 subjects, a validation set of 5 subjects, and a test set of 1 subject. Moreover, each subject appears in an equal number of folds (23, 5, and 1, respectively) for each of the three sets, and with no overlap in subjects between sets of the same fold. The “eye image” module and the “eyelids distance” module were shared across folds. The samples (i.e., 1-min sequences of face images) composing each set are obtained as follows.

For the training set, we adopted a stratified random sampling strategy, where each training epoch consists of an equal number (256) of 1-min sequences randomly drawn from each of five groups (a.k.a. strata). All possible 1-min sequences (of the training set, at a frame level) were divided into five strata based on the number of their four median RTs (noted $m_{i}$ in Section 3.2) that are greater than or equal to 470ms, with this number ranging from 0 to 4 for the five strata, respectively.

For the validation set and test set, we sampled the 1-min sequences that end at the occurrence time of every PVT stimulus (except for the PVT stimuli that occurred within the first minute of the PVT). In this way, the first ground-truth LoD is perfectly time-synchronized with the 1-min sequence. This deterministic sampling strategy leads to an average of about 85 samples per PVT.

We validated the hyper-parameters via random search so as to minimize the average validation loss across the 29 folds. Moreover, while we balanced the training sets (at an epoch level) via stratified random sampling, we balanced the validation sets (across folds) by weighting each sample in the *i*th relative entropy loss function (i.e., the *i*th term of the sum constituting the loss function in Equation (4)) based on whether the median RT $m_{i}$ (of the sample) is lower or greater than 470 ms. This results in eight weights (two per timescale, shared across folds) with values that equal half of the reciprocal of the occurrence frequencies at a specific timescale (indexed by *i*), and across folds. Table 3 shows the eight computed occurence frequencies, and the eight resulting weight values.

We trained the 29 drowsiness models (one per fold) using the Adam [34] optimization routine with a first moment coefficient of 0.9, a second moment coefficient of 0.999, a batch size of 32, and a learning rate of 0.0016029. We used dropout [35] with probabilities of 0.35, 0.7, and 0.35 respectively at three positions: (1) right after the concatenation of the second convolutional layer, (2) right after each global pooling layer, and (3) right before each last fully connected layer. We normalized the eyelids distances by subtracting the average eyelids distance computed from the training set (independently for each fold). We augmented the data by randomly swapping (with a probability of 0.5) the right and left sequences of eyelids distances.

## 6. Evaluation of the Performance of Our System

### 6.1. “Eye Image” Module

We evaluated the performance of the “eye image” module on the held-out test set used for evaluating our “eyelids distance” module. We computed the Root Mean Square Error (RMSE) between (1) the true eye positions obtained from the manually-annotated eye landmarks, and (2) the estimated eye positions obtained from the eyelids landmarks of our “eye image” module. We discarded samples with large errors in estimated eye positions, i.e., when the algorithm did not converge. The reason is that, when processing a sequence of face images, we can easily detect such large errors (e.g., with a threshold on the variation in eye positions), and then estimate better eye positions by either interpolating or extrapolating them from the eye positions of other frames.

Following this evaluation scheme, we obtained an RMSE of 1.2 pixels, which is low enough for the eye to be always entirely contained within the eye image.

### 6.2. “Eyelids Distance” Module

We evaluated the “eyelids distance” module performance on the held-out test set composed of 4640 eye images from 70 subjects, and obtained an RMSE of 0.523 pixels.

For purposes of comparison, we also produced the eyelids distances directly from the eyelids landmarks localized by the “eye image” module, scaled them to be referenced in the coordinates of the eye image (rather than those of the face image), and obtained an RMSE of 1.152 pixels on the same held-out test set. This significant difference of a 1.152/0.523=2.2 factor in performance clearly motivates the use of a specialized module, i.e., the “eyelids distance” module, for producing the eyelids distances.

Indeed, face alignment techniques, such as the one used in the “eye image” module, aim at localizing landmarks positioned on the entire face, rather than only those positioned on the eyelids. Because of this, the localization of eyelids landmarks significantly depends on the positions of other landmarks. This inter-landmark dependency is crucial for good coarse localization of the eyelids landmarks, but limits the fine localization of these landmarks since these are few in number (about 20% of all face landmarks). On the contrary, our “eyelids distance” module aims at directly producing an estimate of the eyelids distance from the eye image, which can be efficiently carried out with a CNN.

### 6.3. “Drowsiness” Module

We evaluated the performance by aggregating the results of the 29 test sets, which are computed by their respective trained model, before computing the performance metrics. We did not average the performance metrics across the 29 subjects because (1) the amount of data was not identical for all subjects (some PVTs were missing), and (2) the proportion of fast/slow RTs varied significantly between subjects.

In addition, we discarded, at each timescale *i* independently, the samples with a ground-truth probability of drowsiness $p_{i}$ of 0.5. That is, we only kept the samples whose median RT $m_{i}$ is below 400 ms ($p_{i}=0$, the sample is labeled as alert, the negative class), or above 500 ms ($p_{i}=1$, the sample is labeled as drowsy, the positive class). This discarding resulted, for the 1st, 2nd, 3rd, and 4th timescales respectively, in aggregated (across folds) numbers of alert/drowsy (i.e., negative/positive) samples of 4845/639, 5100/316, 5221/231, and 5345/155.

The obtained results are shown in bold in Table 4. Our system achieved, for the 1st, 2nd, 3rd, and 4th timescales respectively, a specificity (i.e., true negative rate, TNR) of 72.26%, 89.29%, 90.44%, and 94.80%; a sensitivity (i.e., true positive rate, TPR) of 58.69%, 71.84%, 75.76%, and 74.19%; and a global accuracy of 70.69%, 85.45%, 89.82%, and 94.22%. Overall, we observe that all performance metrics increase with the timescale at which the LoD is inferred. The most significant increase in accuracy (of 14.77%) is found between the 1st and the 2nd timescales. These results are consistent with expectations. Indeed, as the timescale increases, the characterization of drowsiness becomes less challenging because (1) the associated ground-truth LoD estimates more accurately the level of drowsiness, and (2) the data-driven features (related to eye closure dynamics) becomes less noisy as they are averaged over a longer time window.

### 6.4. Processing Time of Our System

We evaluated the processing time of each module on a computer equipped with a Nvidia GeForce GTX TITAN X (Maxwell architecture) and an Intel i7-6700. The “eye image” module processes one video frame in 12 ms. The “eyelids distance” module processes one pair of eye images (i.e., the left one and the right one) in 1.2 ms. The “drowsiness” module processes an 1-min sequence of eyelids distances in 2.5 ms, 13 ms, or 62 ms when using 1, 6, or 29 models, respectively. Note that, although the “eye image” module and the “eyelids distance” module have to be applied at each and every new frame, i.e., at 30 times per second, the “drowsiness” module can be applied at a lower rate, e.g., at 10 times per second. In this way, real-time constraints can be satisfied with an adjustable, comfortable margin.

### 6.5. Impact on Performance of the “Multi-Timescale Context”

We study the impact on performance of the “multi-timescale context” (defined in Section 4.3) by training, validating the hyper-parameters, and evaluating the 29 models without this context, i.e., by removing the auxiliary branch that is concatenated into each of the four main branches. We doubled the depth of the first fully connected layer to compensate for the reduced number of parameters.

The results in Table 5 show that the accuracy significantly drops at the 1st timescale (from 70.68% to 61.94%) accompanied with an increase in sensitivity (from 58.69% to 65.26%), and that the sensitivity drops at the 2nd, 3rd, and 4th timescales (by 3.49%, 7.79%, and 3.87%, respectively). This mostly shows that the context (of eye closure dynamics) from the higher timescales is crucial for good performance at the low timescales. This makes sense since a single long blink is more probably associated with a lapse if the driver has experienced long blinks for the last minute than if he has not.

## 7. Comparison by Proxy of Performance between Our System and Related Systems

The comparison of performance with those reported in other studies, i.e., in Table 4, requires some caution. Indeed, as seen in Section 1 and given the lack of a clear consensus, there exists a wide range of approaches to annotating the ground-truth LoD. In particular, some of these annotated ground-truth LoDs are intrinsically more correlated with the image content than others. For instance, García et al. [19] annotate the ground-truth LoD via three experts visually looking for behavioral signs of drowsiness in the face video, which is more correlated with the image content than our ground-truth LoDs annotated by thresholding the RTs of a PVT. It is thus logical that their system achieves higher sensitivity than ours. In addition, the type of task performed by their subjects, i.e., driving on real roads, is also different. Therefore, the comparison between different studies is far from being straightforward.

Fair comparisons imply either, or both, of the following approaches: (1) our system is evaluated on the datasets of other studies; (2) systems of other studies are trained and evaluated on our dataset. The first approach is infeasible since evaluating our system requires RTs to be measured—which is rarely the case, and—even if RTs were measured—the datasets of other studies are typically not publicly available anyway. The second approach makes sense only if the ground truth of drowsiness used to train and evaluate the systems of other studies is the same as our system, which is not the case. To provide comparisons as fair as possible, we use a proxy system, i.e., a system that is representative of the ones of other studies. We developed the proxy system, and evaluated its performance under the same conditions as our system, i.e., on the same dataset, with the same multi-timescale ground truth of drowsiness, and via the same evaluation scheme.

### 7.1. Description of the Proxy System

With the goal of representing a panel of systems of other studies that is as wide as possible, the proxy system adopts a cascade structure where (1) its intermediate representation is a vector of standard ocular features, and (2) its drowsiness model is composed of four linear SVMs (one per timescale). Furthermore, to provide fair comparisons with our multi-timescale system, the vector of standard ocular features is composed of six standard ocular features computed at four timescales (i.e., 5 s, 15 s, 30 s, and 60 s). We used the six following standard ocular features: the mean blink duration, the mean closing duration, the mean closed duration, the mean re-opening duration, the number of microsleeps (defined as blinks with duration above 500 ms), and the percentage of closure below 70% (PERCLOS-70).

More specifically, we extracted the vector of ocular features with the algorithm of Massoz et al. [36] applied to the sequence of eyelids distances produced by our “eyelids distance” module. First, we compute the sequence of maximum eyelids distances with adaptive exponential smoothing. Second, we produce the sequence of normalized eyelids distances, i.e., eyelids distance divided by the maximum eyelids distance. Third, we apply empirically-determined thresholds on the first derivative sequence of normalized eyelids distances to identify the time segments corresponding to each part (i.e., closing, closed, re-opening parts) of each blink. Fourth, we compute the six types of ocular features from the segmented blinks that occurred within four most-recent time windows with durations of 5 s, 15 s, 30 s, and 60 s, resulting in a vector of 24 ocular features. Note that, by feeding, as input, ocular features computed from four time windows, each SVM characterizes drowsiness with “multi-timescale context”.

### 7.2. Training of the Proxy System

We trained each SVM, i.e., each timescale, separately. At each timescale, we trained 29 models following a leave-one-subject-out cross-validation strategy of 29 folds. However, considering the significantly faster training time of SVMs compared to CNNs, we validated the regularization hyper-parameter *C* via an inner leave-one-subject-out cross-validation strategy of 28 folds, i.e., all subjects but the one in the test set of the outer cross-validation. Upon determination of the optimal value of *C*, we trained the final model on all 28 subjects of the training set (of the outer cross-validation).

We obtained all samples of the training, validation, and test sets in the same manner, i.e., by sampling the 1-min sequences that end at the occurrence time of every PVT stimulus (except for the PVT stimuli that occurred within the first minute of the PVT). We discarded samples with a ground-truth probability of drowsiness $p_{i}$ of 0.5, for all three sets and at each timescale *i* independently (as in Section 6.3). We individually normalized each feature so as to be within the range [0,1] for the samples of the training set. We weighted the classes (i.e., alert and drowsy, as in Section 6.3) in the SVM optimization routine with the reciprocal of the number of their occurrence in the training set. We performed training and inference with the LIBLINEAR library [37]. We performed no data augmentation.

### 7.3. Evaluation of the Performance of the Proxy System, and Comparison

We evaluated the performance of the proxy system by aggregating the results of the 29 test sets, which are computed by their respective trained model, before computing the performance metrics. The obtained results are shown in Table 6. The proxy system achieved, for the 1st, 2nd, 3rd, and 4th timescales respectively, a specificity (i.e., TNR) of 64.43%, 78.71%, 81.06%, and 84.49%; a sensitivity (i.e., TPR) of 61.03%, 65.81%, 60.34%, 64.52%; and a global accuracy of 64.03%, 77.97%, 80.18%, and 83.93%.

Overall, the proxy system performs worse than our system, as well as worse than some of other studies (e.g., García et al. [19], and Huynh et al. [23]). This shows that the task of estimating drowsiness defined via responsiveness performance, i.e., median RTs, is not straightforward. The fact that our system outperforms the proxy system demonstrates the appropriateness of using a temporal CNN architecture to process a sequence of eyelids distances so as to characterize drowsiness.

## 8. Combination of Multi-Timescale Decisions

Up to now, we attained the above results and observations by considering the four binary LoDs individually. When considered together, the four LoDs have $2^{4}$ (16) possible outcomes. Interestingly, whereas the (combined) ground-truth LoD takes its value from all of the 16 possible outcomes, the (combined) inferred LoD takes its value only from 5 outcomes: “0000”, “1000”,“1100”, “1110”, and “1111”. This means, that if our system detects drowsiness at one timescale (e.g., 30 s), it will consequently detect drowsiness at all lower timescales (e.g., 5 s and 15 s). As a corollary, it also means that the detection of drowsiness at one timescale (e.g., 5 s) will happen before (or, at worst, at the same time) than the detections at higher timescales (e.g., 15 s and above).

This suggests that our system has “learned” some form of internal timescale hierarchy as a result of the fact that we have trained the four classifiers together. However, it is also possible that this behavior of our system simply stems from the built-in hierarchy of the time windows (of 5 s, 15 s, 30 s, and 60 s) at the global pooling stage of the “drowsiness” module.

One could thus build a unified classifier by adding the binary decisions of each classifier so as to output a combined LoD ranging from 0 to 4 (with the lower levels being more responsive, and the higher ones more accurate). In real-world applications, one can conveniently feed such combined LoD back to the driver and/or to a semi-autonomous driving system. Indeed, when the (combined) LoD reaches 1, the driver would take notice early that he/she might be starting to be drowsy. At this time, the driver should determine the plausibility of drowsiness by answering whether he/she has been driving for a long time, and whether he/she had enough sleep. When the LoD reaches 2–3, drowsiness becomes more and more probable, and the driver can start taking early safety actions. When the LoD reaches 4, drowsiness is most probable, and the driver would have had enough time to decide the best safety actions to take, such as pulling to the nearest rest area to switch drivers, take a 15-min nap, and/or consume a caffeinated beverage [38]. Note that, whereas a driver may become too drowsy to take any safety actions, a semi-autonomous driving system would always be ready to take the actions necessary to prevent any accidents, including autonomously bringing the vehicle to the nearest rest area.

## 9. Conclusions

In this paper, we have presented a new multi-timescale drowsiness characterization system that is novel, data-driven, automatic, real-time, and generic. Our system processes a 1-min face video with three successive modules, extracts data-driven features related to eye closure dynamics at distinct timescales (5 s, 15 s, 30 s, and 60 s), and outputs four binary LoDs with diverse trade-offs between accuracy and responsiveness. We have presented a multi-timescale ground truth of drowsiness (required to train multi-timescale systems) that consists of four ground-truth LoDs based on thresholded, normalized median RTs computed from time windows of different lengths.

We have evaluated our system in controlled, laboratory conditions on 29 subjects via a leave-one- subject-out cross-validation. The results show that our system achieves overall strong performance, with the highest performance (specificity of 94.80%, and sensitivity of 74.19%) at the 4th timescale (of 60 s). We showed that our system outperforms a proxy system based on a vector of multi-timescale, standard ocular features being fed to linear SVMs, which is representative of a wide range of systems found in other studies.

In real-world applications, the driver (or a monitoring system and/or a semi-autonomous driving system) could combine these four estimated LoDs (of increasing accuracy, and of decreasing responsiveness) to assess the driver’s physiological state of drowsiness, and then decide—with full knowledge—to take safety actions such as pulling to the nearest rest area.

## Figures and Tables

**Table 1:** Related work on drowsiness characterization systems. * $N_{s}$ stands for the number of subjects.

Drowsiness Is...	Paper	Drowsiness Dataset			Intermediate Representation	Drowsiness Model
		Acquisition Setting	${N_{s}}^{*}$	Size		
...self-annotated by subjects	Wang and Xu [14]	Driving simulator	16	16 h	Ocular and driving features	ANN
	Ebrahim et al. [15]	Real road & dr. sim.	43	67 h	Ocular (EOG) features	SVM/ANN
...marked positive when line crossings occur	Vural et al. [16]	Driving simulator	4	12 h	Facial expressions features	Logistic regression
	Liang et al. [17]	Real road	16	36 h	Ocular and driving features	Logistic regression
...annotated by trained experts from brain signals						
	François et al. [18]	PVT	24	12 h	Ocular features	Unspecified
...annotated by trained experts from face video	García et al. [19]	Real road	10	30 h	Ocular and driving features	ANN
	Nopsuwanchai et al. [20]	Driving simulator	13	11 h	Sequence of ocular features	HMM
...acted out according to a pre-defined script (non spontaneous)	Weng et al. [21]	Driving simulator	36	9 h 30	Seq. of ocular, mouth, and head pose features	HMM
	Shih and Hsu [22]	Driving simulator	36	9 h 30	Seq. of VGG-16 features	LSTM + CNN
	Huynh et al. [23]	Driving simulator	36	9 h 30	None (end-to-end)	3D-CNN
**...marked positive** **when median RT** **is above 500ms** **(at 4 timescales)**	**Present article**	**PVT**	**29**	**13 h 40**	**Seq. of raw** **eyelids distances**	**Multi-timescale** **temporal CNN**

**Table 2:** Numbers of subjects and samples in the training, validation, and test sets, and from each sub-datasets (MPIE and DD) of the “eyelids distance” module. For each set, horizontal flipping of every eye image doubles the number of samples contained in this table.

		MPIE	DD	Total
**Number of subjects**	**Training set**	242	11	253
	**Validation set**	28	2	31
	**Test set**	67	3	70
**Number of samples**	**Training set**	6438	1090	7528
	**Validation set**	794	182	976
	**Test set**	1924	396	2320

**Table 3:** The computed occurrence frequencies, *f*, and resulting weight values, *w*, that are used to balance the alert/drowsy samples in the average validation loss (across the 29 folds). Note that both *f* and *w* are functions of (1) the timescale index *i*, and (2) whether the median RT $m_{i}$ is lower or greater than 470 ms.

Timescale	Occurence Frequency *f* (%)		Weight Value $w=f^{-1}/2$	
Index *i*	$m_{i}<470$ ms	$m_{i}\geq 470$ ms	$m_{i}<470$ ms	$m_{i}\geq 470$ ms
1	87.29	12.71	0.5728	3.9318
2	92.97	7.03	0.5378	7.1173
3	94.79	5.21	0.5275	9.5810
4	95.75	4.25	0.5222	11.7821

**Table 4:** Classification performance of our system (in bold) compared to those of other studies. The negative class corresponds to the “alert” label, and the positive class to the “drowsy” label.

System	Reported Performance Metric(s)		Results
Wang and Xu [14]	Average recall (3 classes)		56.04%
Ebrahim et al. [15]	TNR; TPR	with SVM model	76.6%; 64%
		with ANN model	75.9%; 65.2%
Vural et al. [16]	Area under the ROC curve (AUC)		0.98
Liang et al. [17]	TNR; TPR; AUC (subject-specific, not -generic)		98%; 67%; 0.92
François et al. [18]	TNR; TPR		80%; 72%
García et al. [19]	TNR; TPR		95.8%; 85.5%
Nopsuwanchai et al. [20]	Visual (4 classes)		-
Weng et al. [21]	F1 score; Accuracy		85.39%; 84.82%
Shih and Hsu [22]	F1 score; Accuracy		82.82%; 82.61%
Huynh et al. [23]	F1 score; Accuracy		87.97%; 87.46%
**Ours**	**TNR; TPR; Accuracy**	**timescale = 5 s**	**72.26%; 58.69%; 70.68%**
		**timescale = 15 s**	**86.29%; 71.84%; 85.45%**
		**timescale = 30 s**	**90.44%; 75.76%; 89.82%**
		**timescale = 60 s**	**94.80%; 74.19%; 94.22%**

**Table 5:** Comparison of the performance of our system with and without “multi-timescale context”. For each timescale, we put in bold the maximum between (1) the performance metrics (TNR, TPR, and accuracy) obtained with “multi-timescale context”, and (2) the ones obtained without.

“Multi-Timescale Context”?	Timescale (s)	TNR (%)	TPR (%)	Accuracy (%)
**Yes**	5	**72.26**	58.69	**70.68**
	15	**86.29**	**71.84**	**85.45**
	30	90.44	**75.76**	89.82
	60	**94.80**	**74.19**	**94.22**
No	5	61.51	**65.26**	61.94
	15	82.94	68.35	82.09
	30	**91.02**	67.97	**90.04**
	60	94.78	70.32	94.09

**Table 6:** Classification performance of the proxy system compared to our system (in bold) and those of other studies.

System	Reported Performance Metric(s)		Results
Ebrahim et al. [15]	TNR; TPR	with SVM model	76.6%; 64%
		with ANN model	75.9%; 65.2%
François et al. [18]	TNR; TPR		80%; 72%
García et al. [19]	TNR; TPR		95.8%; 85.5%
Huynh et al. [23]	F1 score; Accuracy		87.97%; 87.46%
**Ours**	**TNR; TPR; Accuracy**	**timescale = 5 s**	**72.26%; 58.69%; 70.68%**
		**timescale = 15 s**	**86.29%; 71.84%; 85.45%**
		**timescale = 30 s**	**90.44%; 75.76%; 89.82%**
		**timescale = 60 s**	**94.80%; 74.19%; 94.22%**
Proxy system (linear SVMs), based on a vector of standard ocular features	TNR; TPR; Accuracy	timescale = 5 s	64.43%; 61.03%; 64.03%
		timescale = 15 s	78.71%; 65.81%; 77.97%
		timescale = 30 s	81.06%; 60.34%; 80.18%
		timescale = 60 s	84.49%; 64.52%; 83.93%

## Appendix A. Implementation Details and Links for Data, Code, and Trained Models

For producing the reported results, we used CUDA version 8.0.61, CUDNN version 5.1.10, NVIDIA driver 384, Torch7 git commit 5beb83c46e91abd273c192a3fa782b62217072a6, and Ubuntu 16.04.4 LTS. Our drowsiness dataset, code, and trained models are available at http://www.telecom.ulg.ac.be/mts-drowsiness.
